# Implementation barriers for mHealth for non-communicable diseases management in low and middle income countries: a scoping review and field-based views from implementers

**DOI:** 10.12688/wellcomeopenres.15581.2

**Published:** 2020-04-16

**Authors:** Josefien van Olmen, Erica Erwin, Ana Cristina García-Ulloa, Bruno Meessen, J. Jaime Miranda, Kirsty Bobrow, Juliet Iwelunmore, Ucheoma Nwaozuru, Chisom Obiezu Umeh, Carter Smith, Chris Harding, Pratap Kumar, Clicerio Gonzales, Sergio Hernández-Jiménez, Karen Yeates

**Affiliations:** 1 Primary and Interdisciplinary Care, University of Antwerp, Antwerpen, 1000, Belgium; 2 Department of Public Health, Institute of Tropical Medicine, Antwerp, Antwerpen, 1000, Belgium; 3 Global Health Research Office, Queen’s University, Ontario, Canada; 4 Pamoja Tunaweza Research Center, Moshi, Tanzania; 5 Instituto Nacional de Ciencias Médicas y Nutrición Salvador Zubirán, Mexico City, Mexico; 6 Collectivity, Brussels, Belgium; 7 CRONICAS Centre of Excellence in Chronic Diseases, Universidad Peruana Cayetano Heredia, Lima, Peru; 8 School of Medicine, Universidad Peruana Cayetano Heredia, Lima, Peru; 9 University of CapeTown, Capetown, South Africa; 10 Oxford University, Oxford, UK; 11 Department of Behavioral Science and Health Education, St. Louis University Salus Center, Sint Louis, USA; 12 College of Global Public Health, New York University, New York, USA; 13 Faculty of Health Sciences, Queen’s University, Ontario, Canada; 14 ConnectMed, Nairobi, Kenya; 15 Strathmore Business School, Institute of Healthcare Management, Nairobi, Kenya; 16 Medicine, Queen's University, Ontario, Canada

**Keywords:** diabetes, implementation research, Low and middle income countries, mobile health

## Abstract

**Background**: Mobile health (mHealth) has been hailed as a potential gamechanger for non-communicable disease (NCD) management, especially in low- and middle-income countries (LMIC). Individual studies illustrate barriers to implementation and scale-up, but an overview of implementation issues for NCD mHealth interventions in LMIC is lacking. This paper explores implementation issues from two perspectives: information in published papers and field-based knowledge by people working in this field.

**Methods**: Through a scoping review publications on mHealth interventions for NCDs in LMIC were identified and assessed with the WHO mHealth Evidence Reporting and Assessment (mERA) tool. A two-stage web-based survey on implementation barriers was performed within a NCD research network and through two online platforms on mHealth targeting researchers and implementors.

**Results**: 16 studies were included in the scoping review. Short Message Service (SMS) messaging was the main implementation tool. Most studies focused on patient-centered outcomes. Most studies did not report on process measures and on contextual conditions influencing implementation decisions. Few publications reported on implementation barriers. The websurvey included twelve projects and the responses revealed additional information, especially on practical barriers related to the patients’ characteristics, low demand, technical requirements, integration with health services and with the wider context. Many interventions used low-cost software and devices with limited capacity that not allowed linkage with routine data or patient records, which incurred fragmented delivery and increased workload.

**Conclusion**: Text messaging is a dominant mHealth tool for patient-directed of quality improvement interventions in LMIC. Publications report little on implementation barriers, while a questionnaire among implementors reveals significant barriers and strategies to address them. This information is relevant for decisions on scale-up of mHealth in the domain of NCD. Further knowledge should be gathered on implementation issues, and the conditions that allow universal coverage.

## Introduction

Non-communicable diseases (NCDs) such as diabetes, cardiovascular diseases and other chronic conditions are a large burden for societies globally, due to mortality, morbidity and costs ^1– 3^. Effective interventions for prevention and management of major NCD exist, such as screening and early diagnosis, control with essential drugs and self-management support ^4, 5^. Delivery models for these interventions increasingly include digital channels such as mobile phone applications and websites ^6^.

Mobile health (mHealth) has been hailed as a potential gamechanger for NCD management, even in low- and middle-income Countries (LMIC). The technology has great potential to empower patients, health workers and health system managers through applications like self-monitoring devices, electronic information systems and mobile services for follow-up and community support. mHealth tools can improve the performance of health workers through providing online guidelines and referral services ^7^. They also help retain patients in care through reminders and self-management through information and measurement tools ^8– 11^.

The barriers to implementation of mHealth in LMIC are problematic, especially in the domain of NCD management. Many projects remain stuck at the pilot stage and we have limited evidence on effectiveness, cost, and uptake in these settings ^12^. Individual studies point to implementation barriers; however, few publications provide a detailed description of the implementation process, implementation barriers and how they were addressed. An overview of implementation issues for NCD mHealth interventions in LMIC is lacking. The mHealth evidence reporting and assessment (mERA) checklist was developed in response to the observed need for increased clarity in reporting and describing mHealth interventions, especially in LMIC. Adhering to reporting guidelines facilitates successful replicability to appropriate contexts.

This paper addresses the implementation knowledge gap, by exploring implementation issues from two perspectives: information in published papers and field-based knowledge on implementation by people working in this field. Our main research questions was: what are the barriers for implementation and scale-up of mHealth interventions for non-communicable diseases management in low and middle income countries? For this first perspective, we did a scoping review of the literature on evidence in mHealth studies and assessed the reporting on implementation using the mERA checklist. For the second perspective, we collected views from researchers and health workers working in LMIC through a web-based questionnaire. The two methods allow the combination of formal studies and first-hand informal and tacit knowledge. The combination yields a more complete picture of implementation barriers.

## Methods

### Scoping review

To examine implementation issues reported in publications, we performed a scoping review to find studies on mHealth interventions conducted among an adult population in LMIC, with the prime aim to improve detection and management of cardiovascular disease, diabetes, and/or hypertension. We used the WHO mHealth/e-Health reporting guidelines to map how the publications reported on the key elements of implementation ^13^. The studies included were mHealth interventions conducted among an adult population in LMIC.

***Data collection process.*** The primary search terms were: (chronic disease OR non-communicable disease OR cardiovascular disease OR hypertension OR diabetes) AND (mHealth OR mobile health OR mobile). This list was made starting from the WHO digital tools for NCD used in the ‘ *Be He@lthy Be Mobile*’ initiative ^14^ and further refined through iterative approach. The term *treatment* was interpreted in its broadest sense to include management tools, tools enhancing communication between patient and provider, and tools that provide prognostic or diagnostic information, according to the CONSORT-EHEALTH guidelines ^15^. Our search strategy (extended data ^16^) was guided by the formative methodological framework for scoping reviews ^17^. It included papers in English from the databases PubMed, EMBASE, MedLine, Web of Science, CINAHL, Cochrane and Global Health, published in peer-reviewed journals between 2012 and 2017, with a focus on LMICs. The most recent search was conducted in February 2018.

***Study selection process.*** The types of studies included were randomized controlled trials, cross-sectional studies, case control studies, and cohort studies. The studies were required to be published in the English language, in a peer-reviewed journal and be available in full text format. Studies focusing only on prevention were excluded. One author was responsible for screening records (EE), and 6 authors were responsible for determining eligibility and inclusion (EE, KY, JI, CS, COU, UN).

***Data abstraction process***. Study design characteristics, including sample size, outcome and comparison/control group for the intervention and intervention characteristics were abstracted for all studies. A form in Google Sheets was used to chart the data (see underlying data ^16^). Data charting was done independently. Following initial selection according to the primary selection criteria, a post hoc screening compared the included articles against the mERA guidelines ^13^.

***Assessment criteria.*** The mERA checklist was used to assess which implementation information was reported. This checklist was developed by a WHO working group of mHealth experts with the aim ‘to identify a minimum set of information needed to define what the mHealth intervention is (content), where it is being implemented (context), and how it was implemented (technical features) to support replication of the intervention’ ^13^. The core 16-item checklist addresses the following categories/themes for reporting: 1. Infrastructure, 2. Technology Platform, 3. Interoperability/Health information systems (HIS) context, 4. Intervention Delivery, 5. Intervention Content, 6. Usability/content testing, 7. User Feedback, 8. Access of Individual Participants, 9. Cost Assessment, 10. Adoption inputs/Program entry, 11. Limitations for delivery at scale, 12. Contextual adaptability, 13. Replicability, 14. Data Security, 15. Compliance with national guidelines or regulatory statutes, and 16. Fidelity of the intervention.

***Scoping review protocol.*** A plan for the scoping review was developed *a priori* but was not formally translated into a protocol and disseminated.

### Web-based questionnaire

The web-based questionnaire was developed to complement information from published literature with field-based knowledge from a broad group of implementers. In a two-step approach, we selected researchers and health workers working in NCD mHealth field. The first phase was data collection among a purposively selected sample of researchers with hands-on experience in NCD mhealth implementation research and belonging to an international network: the Global Alliance for Chronic Diseases (GACD) ^18^. A second phase of data collection was added to widen the sample population to include more researchers and practitioners involved in implementation of mHealth in LMIC, in order to enrich the experiences with other projects. This was done, after the analysis of the first phase of data collection, through an online flash consultation with an open invitation to participate to an African-based Community of Practice of local health system managers and international mHealth experts via the online platform Collectivity and Global Digital Health Network (Web Annex 2) ^19, 20^. The first phase lasted from Dec 2017–Jan 2018; the second phase from Jan–March 2018.

***Data collection tool.*** For the first phase, a questionnaire was developed by the research team, and reviewed by two independent researchers for relevance, clarity and completeness. The questionnaire comprised 6 questions on the following topics: 1. Domain of intervention based upon Mecheal *et al*. ^21^ (options: improvement of self-management; lifestyle information & health promotion; improvement of quality of care; addressing health system barriers), 2. Implementation barriers and 3. The way they were addressed, 4. Actors engaged, and 5) Priorities to address for scale-up of the project, and 6. General priorities in mHealth and NCD. It was a semi-structured questionnaire with a combination of closed questions (with options to select) and open-ended questions allowing for additional qualitative information. Although the questionnaire was not pre-tested before administration with the target group, the iterative process of approaching the respondents for more clarity allowed for refinement of answers when original questions were not clear. The data were collected by self-completion, followed through email exchange on clarification if information was not clear. For the flash consultation in the second phase, a more general open-ended questionnaire was used (extended data ^16^. This was done to keep the invitation to participate open for a broad response. In the subsequent email exchange with the respondents to the flash consultation, specifications were asked to clarify answers.

***Response rate.*** Researchers/implementers from 8 out of 13 projects (61.5%) responded in the first round. Six researchers/implementers responded in the second round in the second (two were excluded because of insufficient data). A response rate was could not be calculated for the second round because of the open invitation to participate did not capture non-respondents. For most projects (8 out of 12), the questionnaire was answered independently by two researchers/implementers working on the project. The majority of the projects (83.4%) were research projects and two projects (16.6 %) were privately initiated by a telecom operator or a health care provider.

***Data analysis.*** Analysis of the responses was done through a deductive approach led by the first and last author (JVO, KY). The analysis started from pre-identified themes – inspired by mERA and expert guidance from the author. Responses were classified and information was further reviewed for additional themes. Interpretation of the responses was validated through sharing the draft manuscript with respondents. Six out of the 12 respondents contribute to the text with adding detail, examples and clarifications.

### Patient and public involvement

The underlying studies have developed their intervention in a participatory way. The dissemination was arranged for each study separately. Patients were not involved in the design of this study, nor in the recruitment and conduct of the study.

## Results

### Scoping review

**Figure 1.**
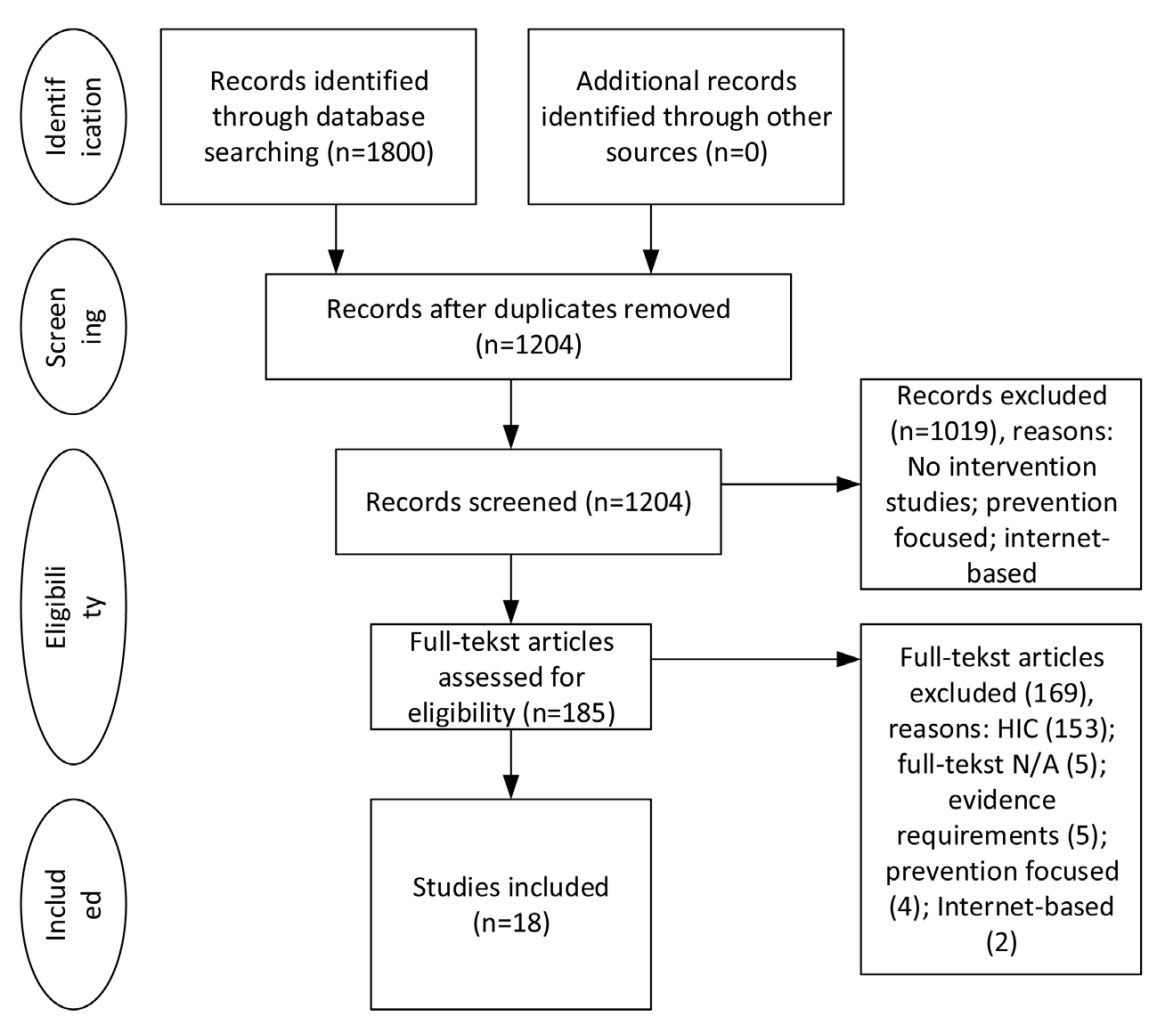
Preferred Reporting Items for Systematic Reviews and Meta-Analyses (PRISMA) flow diagram for the scoping review process.

In total, 16 studies out of 185 papers were included in the final review ( Figure 1). The studies originated from a variety of countries including: India, Pakistan, China, Tibet, Mexico, Iran, Cambodia, Democratic Republic of Congo, Philippines, Tanzania, Bangladesh, Chile, and Malaysia. More than half of the studies utilized SMS (text messaging) as their main intervention tool. Most of these were patient-centered interventions. One study targeted health providers using a mobile decision support platform. While the majority of the interventions focus on diabetes as the core condition, others addressed heart failure, acute coronary syndrome follow-up, cholesterol risk assessment, foot ulcers, and drug adherence. The majority of studies were relatively small with sample sizes ranging from 48 to 3393 participants.

In terms of effectiveness, 15 studies showed improvements in clinical patient related end-points. Table 1 summarizes the mERA checklist items reported on by each of the studies included in the scoping review. In addressing the mERA checklist items, the outcomes were variable with 6 studies addressing mHealth infrastructure domains and 8 addressing characteristics of the technology platform ^22– 29,^. Only 4 studies addressed interoperability ^23, 25, 30, 31^ and 9 addressed usability ^23– 28, 30, 32^. For the intervention itself, 16 studies address intervention delivery and 12 addressed intervention content. For the remaining checklist items, 4 studies addressed user feedback ^25, 27, 30, 32^, 4 address the domain of the individual participant ^23, 25, 26, 30^, and only 2 made any reference to study intervention costs ^23, 26^. Finally, 3 studies included brief information on the adoption of the program ^22, 29, 33^ and 3 studies addressed issues regarding limitations for scaling ^23, 30, 34, 35^. The few papers that commented on barriers noted the participation to be low, and lowering over time ^23, 30^. Interventions that combined SMS with personal phone calls or with interactive feedback on patients input reported on patient satisfaction ^34, 36^. A lack of information on cost is a barrier to further scale-up.

**Table 1.** Scoping Review Results – Applying the mERA checklist to studies of mHealth interventions for management of diabetes, hypertension and cardiovascular disease in LMIC. LMIC = Low and Middle Income Countries. SMS = Short Messages Services.

Reference	Title	Country	Infrastructure	Technology/ Platform	Interoperability	Intervention Delivery	Intervention Content	Usability/Content Testing	User Feedback	Access of Individual Participants	Cost Assessment	Adoption Inputs and Program Inputs	Limitations for Delivery at Scale
Shahid *et al*., 2015	Mobile phone intervention to improve diabetes care in rural areas of Pakistan: a randomized controlled trial	Pakistan				X	X						
Patnaik *et al*., 2015	Mobile Based Intervention for Reduction of Coronary Heart Disease Risk Factors Among Patients with Diabetes Mellitus Attending a Tertiary Care Hospital of India	India				X							
Tian *et al*., 2015	Cluster-Randomized, Controlled Trial of a Simplified Multifaceted Management Program for Individuals at High Cardiovascular Risk (SimCard Trial) in Rural Tibet, China, and Haryana, India	Tiber, China, India				X	X						X
Haddad *et al*., 2014	A Feasibility Study of Mobile Phone Text Messaging to Support Education and Management of Type 2 Diabetes in Iraq	Iraq				X	X						
Kamal *et al*., 2015	A randomized controlled behavioral intervention trial to improve medication adherence in adult stroke patients with prescription tailored Short Messaging Service	Pakistan				X	X						X
Kiselev *et al*., 2012	Active ambulatory care management supported by short message services and mobile phone technology in patients with arterial hypertension	Russia			X								
Naghibi *et al*., 2015	Analyzing Short Message Services Application Effect on Diabetic Patients’ Self-caring	Iran		X		X	X	X					
Mmbali *et al*. 2017	Applicability of structured telephone monitoring to follow up heart failure patients discharged from Muhimbili National Hospital, Tanzania.	Tanzania	X	X		X		X	X				
Sadeghian *et al*. 2017	Application of short message service to control blood cholesterol: a field trial.	Iran		X		X		X			X		
Anzaldo- Campos *et al*. 2016	Dulce Wireless Tijuana: A Randomized Control Trial Evaluating the Impact of Project Dulce and Short-Term Mobile Technology on Glycemic Control in a Family Medicine Clinic in Northern Mexico.	Mexico	X	X	X	X		X	X	X			
Khonsari *et al*. 2015	Effect of a reminder system using an automated short message service on medication adherence following acute coronary syndrome	Malaysia	X	X		X	X			X		X	
Peimani *et al*. 2016	Effectiveness of short message service- based intervention (SMS) on self-care in type 2 diabetes: A feasibility study.	Iran	X	X			X	X					
Islam *et al*. 2015	Effects of Mobile Phone SMS to Improve Glycemic Control Among Patients With Type 2 Diabetes in Bangladesh: A Prospective, Parallel-Group, Randomized Controlled Trial	Bangladesh	X	X	X	X	X	X		X	X		X
Van Olmen *et al*. 2017	The effect of text messages to support diabetes self-management in developing countries - A randomised trial	DR Congo, Cambodia, Philippines	X	X	X	X	X	X	X	X		X	X
Hassan *et al*. 2017	Mobile Phone Text Messaging to Improve Knowledge and Practice of Diabetic Foot Care in a Developing Country: Feasibility and Outcomes.	Jordan											
Celik *et al*. 2015	Using Mobile Phone Text Messages to Improve Insulin Injection Technique and Glycaemic Control in Patients with Diabetes Mellitus: A Multi-Centre Study.	Turkey											

The few papers that commented on barriers noted the participation to be low, and lowering over time ^23, 30^. Interventions that combined SMS with personal phone calls or with interactive feedback on patients input reported on patient satisfaction ^34, 36^. A lack of information on cost is a barrier to further scale-up.

### Questionnaire

Respondents from 12 projects, all of which were implemented in LMIC, answered to the questionnaire. The interventions were comparable, mostly focusing on health education, self-management and quality of care (and or a combination of these aims.) ( Table 2). Seven projects mentioned the intention to address health systems barriers, such as data collection or supply chain management (extended data ^16^). The projects from the first round were research projects in public health care settings (the supply side), whereas two other projects were private entrepreneurial initiatives that focused on the user (demand side). Many projects shared common barriers and also mentioned similar strategies to address them. Effective strategies across the projects were the ongoing engagement with patients and the adaptations in the way of delivering the message.

**Table 2.** Projects in the survey and intervention domains. NCD – non-communicable disease.

	Country	NCD	self-management	Lifestyle, health promotion	quality of care	health system barriers	Web link or reference
Systematic Medical Assessment, Referral and Treatment for Diabetes care in China using Lay Family Health Promoters - SMART Diabetes	China	Diabetes	x	x	x	x	https://www.gacd.org/ research-projects/ diabetes/dm02
Evaluation of a pilot project to prevent diabetes in the workplace using information technology	Mexico	Diabetes			x		https://www.gacd.org/research-projects/diabetes/dm09
Development of an interactive social network for metabolic control of patients with diabetes	Mexico	Diabetes	x				https://www.gacd.org/research-projects/diabetes/dm10
Development and validation of a software to facilitate medical treatment of the patient with type 2 diabetes	Mexico, USA	Diabetes	x			x	https://www.gacd.org/research-projects/diabetes/dm11
SMS supporting treatment for people with type 2 diabetes	Malawi, South Africa	Diabetes	x	x		x	https://www.gacd.org/research-projects/diabetes/dm12
The Bangladesh D-Magic Project	Bangladesh	Diabetes		x			37
Implementation of foot thermometry and SMS to prevent diabetic foot ulcer	Peru	Diabetes	x	x	x		38
Tailored Hospital-based Risk Reduction to Impede Vascular Events after Stroke (THRIVES)	Nigeria	Cardiovascular	x	x		x	https://www.ncbi.nlm.nih.gov/pubmed/25042605
CommCare	Haiti	Non-specific		x			39
Guidelines Adherence in Slums Project	Kenya	Non-specific					40
Mind Tale	Bangladesh	Mental heath					41
ConnectMed Kenya	Kenya	Non-specific					42

**Figure 2.**
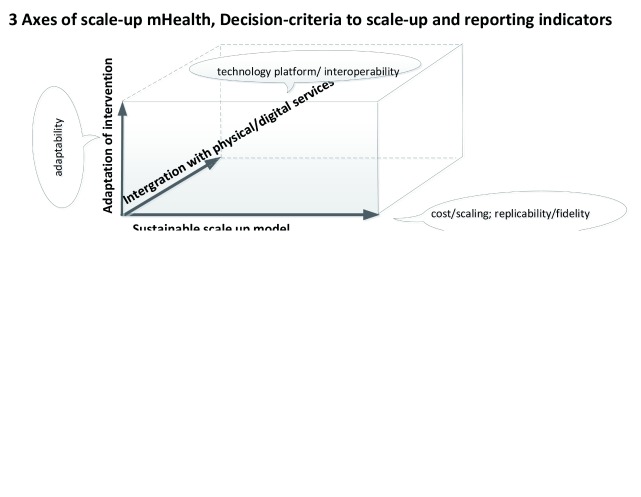
Mapping of axes of decision-making for implementation of mHealth for non-communicable diseases (NCD) (cube), the field views on decision criteria (bottom table) and information from literature studies listed along WHO mHealth Evidence Reporting and Assessment (mERA) items (call-out balloons).

The findings from the survey identified important domains for decision making in the implementation and scale-up of mHealth interventions: 1) reviewing the need for adaptation of the intervention; 2) integrating the mHealth intervention with other digital systems and with the physical health care process; and; 3) designing sustainable scale-up models. We visualized these three domains as three axes of scale-up ( Figure 2). Respondents also identified criteria for decision-making for scale-up of NCD interventions: (a) age, literacy, impairments, expectations and financial means of end users; (b) objective of the intervention (education, self-management, quality of care, access to treatment or follow-up); (c) actors involved (patients, caregivers, managers, health workers, pharmacies, etc.); (d) resources and organization of the health system (treatment and support options, patient records, access and quality); and (e) socio-economic cultural context (behavioral norms, inequalities and gender roles). Figure 2 integrates findings and recommendations of scale-up of mHealth NCD management interventions: the 3-dimensional box shows dimensions on which to act in the scale-up phase; the decision criteria below the box provide 5 aspects to assess in decisions how to scale up; the call-out balloons indicate the indicators to evaluate and report, in accordance to the mERA guidelines.

### Barriers and how they were addressed

*NCD disease related barriers* included age, complications leading to impaired physical or mental functioning, the natural progression of disease and disease-related perceptions. Several respondents mentioned that the highly prevalence of NCD among elderly and among people of lower socio-economic status, led to the combination of low digital literacy and low health literacy. This meant that uptake and engagement of people required additional training and guidance to allow familiarization and growing awareness on health issues. Two projects explicitly mentioned that proactive involvement and follow-up of caregivers and assisting patients improved utilization. They were encouraged to read messages and take them along in their caregiving. Solutions mentioned in two other projects were to change the transmission mode, from SMS to voice messaging and pictograms.

Disease perceptions shaped the expectations of mHealth interventions. Mental health projects reported stigma as a barrier for uptake, which requires attention from the start. Although generally, there was an interest in information on NCDs in LMIC, people also perceived many psychological, physical and cultural barriers to change behavior that could not be changed through the intervention. Low demand and declining engagement with mHealth interventions with a behavior change objective was frequent, even when services were free of charge to patients. Respondents mentioned an average time of engagement of 9 months. To increase patients’ utilization at start, health promotion campaigns addressing stigma were combined with the marketing of an intervention. To maintain engagement over time, some projects ensure personal contact with end-users at regular times, as a form of extrinsic motivation, or to adapt the intervention.

*Barriers related to the mobile intervention.* Health education given via a mobile device required adaptation of the content to the delivery channel. Health education on NCDs and the inclusion of motivations techniques is an area in development and still very limited in many health care settings in LMIC. The translation from personal motivational coaching to a mobile application included an additional challenge in such a context, since the content, the stratification, and timing of information need to be planned ahead in small bits of information with little options for interactivity. Although there were many apps for smartphones, quality varied and they are not always relevant for people in LMIC. The development of the process algorithm and of the motivational messages was typically done in consultation with medical doctors, experts in health behavior, telecom experts, and patients and local health workers. Only one project mentioned involving people from the Ministry of Health. It took on average one year, including testing and validation. Patient-oriented interventions reached patients in their routine of daily life, at work or at home, i.e. beyond the usual setting between health worker and patient. They were mixed with a range of other messages and adverts that attract the attention and might clash with the culture or prevailing ideas in other settings. The amplitude and diversity might lead to message fatigue and dilute the effect of messages.

*Technical barriers* mentioned related to the network provider and operating system, the hardware and software and the linking of different digital systems. The variety of network providers and telecom operating structure across regions made the optimal choice for a platform of data-management and providers difficult, especially in the highly commercialized and volatile telecom sector in many LMIC. The gradual linkage of the project with other functions, or the adaptation of delivery mode (message to voice) usually required adaptations to the technical architecture and renegotiations. Only a few projects have managed to link the project software with existing health management information system or to electronic medical records. The case studies showed the gradation in complexity, from one to multiple actors being able to use the same system. Some respondents mentioned that the involvement of different care providers and departments and the impossibility of allowing interaction between different information systems.

Phone related problems related to people having multiple phones, people switching providers, and phones being turned off. These restrained end users receiving or reading messages sent by the project. Respondents mentioned that tracking systems that monitor delivery of messages and automatically resend, are useful. This software was often embedded in telecom software, and sometimes within software available in open source format. Projects could build upon software that is available from other settings or an open source software platform, but they still required the technical competency to adapt to specific project needs and local users, for instance to allow software to run on old-fashioned smartphones and to adjust websites to low bandwidth internet. While a tablet had more options for intervention design, respondents told us that many users prefer a phone because of the battery life and usability.

*Health care services* barriers mentioned relate to the lack of resources, access to care and integration of mHealth interventions into health care processes including the resistance of health professionals to change. The lack of technical possibilities to link digital systems with each other led to interventions set up in a fragmented fashion, increasing the workload for health workers. Two respondents mentioned that a joint assessment of workflow and workload with health workers increased acceptance, and continuing education and feedback maintained their commitment. Other respondents mentioned that though discussions with health authorities on the need for upgrading public health facilities and through partnering with other health care providers, pharmacies and community organizations, their mHealth project served as a means to expose the unmet need for NCD interventions and could thus contribute to a change process to increase resources for NCDs management and access to care.

*Contextual barriers* mentioned included security, gender differences and social, economic and cultural factors, including limited access to internet of mobile device. One respondent said that informing participants that project phones contained tracking software further prevented theft. In another project, social and gender inequities in access to mobile phones were addressed by encouraging the common use of phones and by sharing of message content, for instance by message delivery at dinner times and encouraging using the speakerphone. This implementation format meant that the format of messages needed adaptation to a common audience, at the cost of personalization. Several respondents mentioned that timing of messages influences the susceptibility of people, so supply-driven interventions need to consider people’s daily routines.

*Regulatory barriers* reported were the lack of clarity on digital health regulations in many countries, which led to continued negotiation in some project. This barrier was more outspoken for the entrepreneurial projects. The stakeholders that were mentioned were the government, medical professional associations and telecom regulators. Medical professional associations in South Africa took the position not to allow mobile consultations without face-to-face contact, which led the project to consider another country. Telecommunications regulators also influenced the delivery options of mHealth interventions, through their policies on number masking, reverse billing and spam filters. Reverse billing (to reduce costs for end-users) needs the operator to allow for special short codes in the system. Sending messages in bulk led to numbers being identified as ‘spam’ or ‘number unknown’, leaving users to not see or not to recognize the message as from their provider.

### Priorities for scale-up of interventions

When asked for the conditions for further scale-up of their intervention, respondents mentioned the priorities: having a human resource plan, a financing or business plan and addressing knowledge gaps. According to answers, a human resource plan should include capacity for process management (interaction with end users and monitoring), for software maintenance and digital health information management, and for evaluation cycles with feedback from users – to ensure relevance and quality on the longer term. Answers on a financing plan, included different options for financing models that would allow for scale-up: direct payment by end-users, contributions from the governments or other parties such as non-governmental organizations, inclusion in health insurance schemes or linking with other services for which there is a large demand. The respondents from research projects mentioned aiming to keeping cost for end-users low, whereas initiatives started as a business began with generation of demand from people able to pay such a mobile money service. Some research projects reported to seek for social enterprises to partner with, trying to bridge both worlds. A project that targeted primary care providers applied a business-to-business model, in which clinics pay a subscription to utilize the intervention. To attract customers, they linked the mHealth session to education sessions about quality of care, which increased the demand for the mHealth. While mixed models of private and public mHealth initiatives are rapidly evolving throughout the world, the need for regulation and for standards of quality also becomes more important. The third priority area mentioned was that of knowledge gaps that hamper scale up. The limited evidence of behavior change messages for diet and physical activity in LMIC, and of how to address comorbidities were mentioned. Respondents mentioned the potential of sharing hands-on implementation knowledge in user-friendly and open ways, via interactive web platforms such as the Global Digital Health Network.

## Discussion

This paper provides an overview of a literature and a field-based view on the challenges to implement and scale up mHealth for NDCs in LMIC. Our findings show that SMS messaging is a dominant mHealth tool for patient-directed of quality improvement interventions in LMIC, and that publications report little on the health system and on context conditions for implementation and scale-up, such as legal regulations and cost little on implementation barriers. The field-views of implementers collected in the web-survey reveals significant barriers and strategies to address them. The main challenges relate to health service organization and cultural context related to mHealth interventions. This information is relevant for decisions on scale-up of mHealth in the domain of NCDs. The combination of the scoping review and the survey information have resulted in a map of decision axis for scale-up of mHealth and an overview of NCD-specific decision-criteria.

The information in this paper echoes implementation challenges from other papers ^43– 45^ but they add an NCDs perspective. The two systematic reviews on *impact* of mHealth on NCDs show modest and variable results ^9, 46^; and the scoping review in this paper points to implementation information being a missing link. The survey findings shed more light on such implementation challenges. These include: age and functional impairments are barriers to utilization; behavior change is a complex process and people might have low expectations of the benefit of an mHealth intervention which lowers the uptake especially in the long term; chronic disease management frequently involves multiple actors who don’t share the same information; the variation in network providers complicates universal access to the intervention.

*Methodological considerations*. The information for this paper was collected through a scoping review and through a two-stage web survey among researchers and implementors. This combination allowed explicit and published knowledge to be combined with more informal resources. The scoping review was limited by the facts that many of the studies were published prior to the development of the mERA checklist. Careful reading and classification yielded a lot of information addressing the mERA themes nevertheless. The scoping literature excluded studies with an exclusive focus on prevention, such as SMS in the general population for behavior change ^33^. Some of such studies might have illustrated similar or additional light on implementation barriers. The limitations of the survey entail the selective group of interviewees in the first round, narrowing the scope of projects. The first and last author who evaluated the responses of the survey are part of the GACD network themselves and knew many respondents of the first round personally. This is likely to have increased the response rate. It might also have led to a selection bias (people knowing the authors being more likely to respond), and to a response bias (likeminded answers). We estimate the latter bias to have been outweighed by the fact that respondent in the first step survey have taken time to formulate in-depth and qualitative responses. The interpretation of responses by the authors might have been colored by the authors’ own experiences. The widening of the survey via Collectivity (thecollectivity.org) and related platforms widened the scope and yielded new perspective. The 2-step approach exposed the difference between research projects and projects that started from an entrepreneur perspective. The informal resources were not checked by other sources such as formal study reports, but for most projects, the questionnaire was completed 2 times, independently by 2 researchers/implementers. Responses were checked for consistency and complementarity. The informal aspect of the data collection has lowered the barrier for participation and the responses show openness to sharing, also on negative results. The field-based stories add a realistic view on implementation and expectations of mHealth for NCDs in LMIC.

This view can support implementation and scale-up of mHealth for NCDs in LMIC. The expertise in behavior change strategies for NCDs in LMIC is limited, and the evidence on mobile health is still being developed. An open-source database with message sets that have been validated could contribute to global knowledge creation, dissemination and facilitate implementation. Ongoing engagement with patients (experts) is essential to understand their needs and perceived benefits, to respond to and stimulate the demand of users. Models that stimulate interaction instead of mere information could increase ongoing engagement and develop competencies for self-care. The progressive nature of NCDs and the involvement of multiple actors over time means that interventions should allow for iterative design, in which the intervention can be adapted and in which integration with other digital and physical health services is possible.

Although literacy will increase over time, the societal inequities in education, in health and in access to resources, will remain. The barriers to health education, motivational strategies and communication in LMIC, need additional study. There will be an increasing mix of private and public business models in the ongoing scale-up initiatives. This indicate the need for the building of a knowledge base on the policy, legal, financial and cultural conditions that allow equitable access to these new interventions and benefits for those in most need in LMIC.

### Ethics

The studies which are included in the review and in the survey have been subject to approval by ethics committee. The survey presents aggregate data that was limited to describing research results from an array of funded research projects. We do not have any human subjects’ data in the study or analyses, and thus we did not seek ethical approval. All participating projects however, received ethical clearance from their respective institutions and other local authorities (e.g. Ministries/Municipalities) to conduct their own studies. All respondents in the survey gave written informed consent to participate in the survey and were informed about how the data will be used in a publication. The data are not used for any other purpose.

## Strengths and limitations of this study

- The strength of this paper is the combination of explicit and published knowledge with more informal resources through a web-based questionnaire which led to sharing of also negative experiences.
- The scoping review was comprehensive; however, a protocol was not published prior to conducting the review.
- The selection of interviewees for the first round of questionnaires was narrow, limiting the scope and generalizability of findings. The additional second round of an open web-based survey enlarged the scope, although the response rate remained low.
- The field-based views in this paper are not published in main literature but relevant for future implementation of mHealth interventions for NCDs in LMIC

## Data availability

### Underlying data

Open Science Framework: Implementation barriers for mHealth for non-communicable diseases prevention and management in low and middle income countries: a survey among implementers. https://doi.org/10.17605/OSF.IO/MR8Y4 ^16^

This project contains the following underlying data:

- mHealth interventions for NCD prevention and control in LMIC_20180108_pseudomised.xlsx (Results of Survey round 1)
- Flash consultation mhealth NCD_results_20180118_pseud_reedit.docx (Results of Survey round 2, flash consultation)

### Extended data

Open Science Framework: Implementation barriers for mHealth for non-communicable diseases prevention and management in low and middle income countries: a survey among implementers. https://doi.org/10.17605/OSF.IO/MR8Y4 ^16^

This project contains the following extended data:

- Annex2a en b.pdf (Survey questionnaires)

Data are available under the terms of the Creative Commons Zero "No rights reserved" data waiver (CC0 1.0 Public domain dedication).
