# Supplementary material for: Implementation barriers for mHealth for non-communicable diseases management in low and middle income countries: a scoping review and field-based views from implementers
Source: Wellcome Open Res. 2020 Apr 16;5:7. Originally published 2020 Jan 14. [Version 2] doi: 10.12688/wellcomeopenres.15581.2 (PMC7194478; doi:10.12688/wellcomeopenres.15581.2)
Supplement: Peer review history

# Peer review: Reviewer response for version 2

**DOI:** 10.21956/wellcomeopenres.17360.r38452

**Published:** 2020-04-22

**Authors:** Ranjani Harish

**Affiliations:** 1 WHO Collaborating Center for Prevention and Control of Noncommunicable Diseases & IDF Centre of Excellence in Diabetes Care, Madras Diabetes Research Foundation and Dr. Mohan's Diabetes Specialties Center, Chennai, India

**Comments to the Authors:**

**General Comments:**  The main aim of the study was to explore barriers for implementation and scale-up of mHealth interventions for non-communicable diseases prevention and management in low and middle income countries.

*Authors:* Indeed, this is our main research question as stated in the last paragraph of the introduction. As explained further down, we have remove the work prevention from the title and the focus

*Re-Reviewer’s comments:* Done

**Specific Comments:**

- It would be more appropriate to give strengths and limitations at the end of the paper.

*Authors* *: We agree with you and have subsequently moved this section to the end of the paper, after discussion.*

*Re-Reviewer’s comments: *Done

- Scoping review section under methods needs to be rephrased. Write the key words first. What is the source of current mHealth/eHealth reporting guidelines?

*Authors:* Thank you for this advice. We have indeed rephrased this section according to your suggestions. The source of the current guidelines is the Mera checklist, also explained in the data abstraction process. We included the reference at the right place in the text *.*

*Re-Reviewer’s comments:* Done

- In scoping review section of results it is mentioned that “ *In total, 18 studies out of 185 papers were included in the final review (Figure 1). The studies originated from a variety of countries including: India, Pakistan, China, Tibet, Mexico, Iran, Cambodia, Democratic Republic of Congo, Philippines, Tanzania, Bangladesh, Chile, and Malaysia.*” This shows that studies from some non LMICs and MICs were also selected. This is contradictory to the main aim of the study. Please explain. Also, scoping review section of methods states that *“The key elements of studies included were mHealth interventions conducted among an adult population in LMICs.”*

*Authors:* the main aim of the study relates to barriers in low and middle income countries, which also includes middle income countries. We chose not only to include low income countries, because Middle Income Countries still face similar challenges relating to health system and context, despite having more money. The author is of course right to correct us that Chile is a HIC, and so is Finland. We have omitted both studies. The numbers in the results section have been adjusted.

*Re-Reviewer’s comments:* Agree

-  According to world bank report on economies (2016-17, 2017-18, 2018 – 19) Chile belongs to high income group and China and Mexico to upper middle income group.

*Authors: *see comment above

*Re-Reviewer’s comments:* Please remove the names of these two countries from second line of the scoping review section of results also.

- As per the methods, the authors have included papers in the time gap of 2012-2018. However, the 18 studies included are from 2015-2017. Even then an important large scale text messages based study from India by Pfammatter *et al. *(2016) ^1^, has been missed out.

*Authors:* We appreciate the suggestion of the reviewer about the study in India. This study was not missed in the screening. It was however excluded because it is largely focused on prevention. We realise that the title of our paper is misleading as our studies were focused on management of NCD. The implementation barriers also focus partly on the integration with health services. We have omitted the word ‘prevention’ from the title. We realise that this is a limitation of the study. We added a sentence about this in the discussion. We mention in that part also other studies, such as the one suggested by the reviewer.

*Authors:* although the time gap was indeed larger, most studies selected were from a narrow period.

*Re-Reviewer’s comments: *carried out

- Include the reason for excluding 1019 studies in the flowchart.

 
*Authors: *we did so. It was not possible to quantify the reasons, since this was not recorded.

*Re-Reviewer’s comments: * Done

- Figure 2 is difficult to interpret on its own and has not been explained in detail. It is a complex figure to follow.

*Authors:* We have simplified the figure and provided additional text to explain it better. 

*Re-Reviewer’s comments: *Done

- Kindly check the paper for grammatical errors. A few are listed below for your reference:
  - In the second line of study selection section; word “for” is missing *–“6 authors were responsible determining eligibility and inclusion”.*
  - In the fourth line of the same section; it should be “was” instead of “are”, as the whole paragraph is written in the past tense, except for this sentence. *“The types of studies included are randomized controlled trials, crosssectional studies, case control studies, and cohort studies.”*
  - In first line of page 4, it should be “tested” in place of “testing” – *“Although the questionnaire was not pre-testing before administration with the target group,”*

*Authors* *: Thank you for pointing out these errors. We have carefully gone through the manuscript again and re-edited for proper English.*

*Re-Reviewer’s comments:* Carried out

Is the work clearly and accurately presented and does it cite the current literature?

Partly

If applicable, is the statistical analysis and its interpretation appropriate?

Partly

Are all the source data underlying the results available to ensure full reproducibility?

Partly

Is the study design appropriate and is the work technically sound?

Partly

Are the conclusions drawn adequately supported by the results?

Partly

Are sufficient details of methods and analysis provided to allow replication by others?

Partly

Reviewer Expertise:

Epidemiology and Prevention of obesity and type 2 diabetes in the young and adults. Diabetes prevention based implementation programs at different settings such as worksites, schools and community. mhealth and diabetes. Built environment and Physical activity in adolescents. Qualitative research.

I confirm that I have read this submission and believe that I have an appropriate level of expertise to confirm that it is of an acceptable scientific standard.

# Peer review: Reviewer response for version 1

**DOI:** 10.21956/wellcomeopenres.17062.r37599

**Published:** 2020-02-07

**Authors:** Ranjani Harish

**Affiliations:** 1 WHO Collaborating Center for Prevention and Control of Noncommunicable Diseases & IDF Centre of Excellence in Diabetes Care, Madras Diabetes Research Foundation and Dr. Mohan's Diabetes Specialties Center, Chennai, India

**Comments to the Authors:**

**General Comments:**  The main aim of the study was to explore barriers for implementation and scale-up of mHealth interventions for non-communicable diseases prevention and management in low and middle income countries.

**Specific Comments:**

- It would be more appropriate to give strengths and limitations at the end of the paper.
- Scoping review section under methods needs to be rephrased. Write the key words first. What is the source of current mHealth/eHealth reporting guidelines?
- In scoping review section of results it is mentioned that “ *In total, 18 studies out of 185 papers were included in the final review (Figure 1). The studies originated from a variety of countries including: India, Pakistan, China, Tibet, Mexico, Iran, Cambodia, Democratic Republic of Congo, Philippines, Tanzania, Bangladesh, Chile, and Malaysia.*” This shows that studies from some non LMICs and MICs were also selected. This is contradictory to the main aim of the study. Please explain. Also, scoping review section of methods states that *“The key elements of studies included were mHealth interventions conducted among an adult population in LMICs.”*
-  According to world bank report on economies (2016-17, 2017-18, 2018 – 19) Chile belongs to high income group and China and Mexico to upper middle income group.
- As per the methods, the authors have included papers in the time gap of 2012-2018. However, the 18 studies included are from 2015-2017. Even then an important large scale text messages based study from India by Pfammatter *et al. *(2016) ^1^, has been missed out.
- Include the reason for excluding 1019 studies in the flowchart.
- Figure 2 is difficult to interpret on its own and has not been explained in detail. It is a complex figure to follow.
- Kindly check the paper for grammatical errors. A few are listed below for your reference:
  - In the second line of study selection section; word “for” is missing *–“6 authors were responsible determining eligibility and inclusion”.*
  - In the fourth line of the same section; it should be “was” instead of “are”, as the whole paragraph is written in the past tense, except for this sentence. *“The types of studies included are randomized controlled trials, crosssectional studies, case control studies, and cohort studies.”*
  - In first line of page 4, it should be “tested” in place of “testing” – *“Although the questionnaire was not pre-testing before administration with the target group,”*

This paper can be accepted for indexing after the authors have reworked the paper based on the suggestions given above.

Is the work clearly and accurately presented and does it cite the current literature?

Partly

If applicable, is the statistical analysis and its interpretation appropriate?

Partly

Are all the source data underlying the results available to ensure full reproducibility?

Partly

Is the study design appropriate and is the work technically sound?

Partly

Are the conclusions drawn adequately supported by the results?

Partly

Are sufficient details of methods and analysis provided to allow replication by others?

Partly

Reviewer Expertise:

Epidemiology and Prevention of obesity and type 2 diabetes in the young and adults. Diabetes prevention based implementation programs at different settings such as worksites, schools and community. mhealth and diabetes. Built environment and Physical activity in adolescents. Qualitative research.

I confirm that I have read this submission and believe that I have an appropriate level of expertise to confirm that it is of an acceptable scientific standard, however I have significant reservations, as outlined above.

## References

[1] : mHealth Intervention to Improve Diabetes Risk Behaviors in India: A Prospective, Parallel Group Cohort Study. *Journal of Medical Internet Research*.2016;18(8) : 10.2196/jmir.5712 10.2196/jmir.5712 PMC499216927496271

# Response

**Published:** 2020-03-23

**Authors:** Josefien van Olmen

**Affiliations:** University of Antwerp, Belgium

**Comments to the Authors:**

**General Comments:**  The main aim of the study was to explore barriers for implementation and scale-up of mHealth interventions for non-communicable diseases prevention and management in low and middle income countries.

*Authors:* Indeed, this is our main research question as stated in the last paragraph of the introduction. As explained further down, we have remove the work prevention from the title and the focus

**Specific Comments:**

- It would be more appropriate to give strengths and limitations at the end of the paper.

*Authors* *: We agree with you and have subsequently moved this section to the end of the paper, after discussion. *

- Scoping review section under methods needs to be rephrased. Write the key words first. What is the source of current mHealth/eHealth reporting guidelines?

*Authors:* Thank you for this advice. We have indeed rephrased this section according to your suggestions. The source of the current guidelines is the Mera checklist, also explained in the data abstraction process. We included the reference at the right place in the text *.*

- In scoping review section of results it is mentioned that “ *In total, 18 studies out of 185 papers were included in the final review (Figure 1). The studies originated from a variety of countries including: India, Pakistan, China, Tibet, Mexico, Iran, Cambodia, Democratic Republic of Congo, Philippines, Tanzania, Bangladesh, Chile, and Malaysia.*” This shows that studies from some non LMICs and MICs were also selected. This is contradictory to the main aim of the study. Please explain. Also, scoping review section of methods states that *“The key elements of studies included were mHealth interventions conducted among an adult population in LMICs.”*

*Authors:* the main aim of the study relates to barriers in low and middle income countries, which also includes middle income countries. We chose not only to include low income countries, because Middle Income Countries still face similar challenges relating to health system and context, despite having more money. The author is of course right to correct us that Chile is a HIC, and so is Finland. We have omitted both studies. The numbers in the results section have been adjusted.

-  According to world bank report on economies (2016-17, 2017-18, 2018 – 19) Chile belongs to high income group and China and Mexico to upper middle income group.

*Authors: *see comment above

- As per the methods, the authors have included papers in the time gap of 2012-2018. However, the 18 studies included are from 2015-2017. Even then an important large scale text messages based study from India by Pfammatter *et al. *(2016) ^1^, has been missed out.

*Authors:* We appreciate the suggestion of the reviewer about the study in India. This study was not missed in the screening. It was however excluded because it is largely focused on prevention. We realise that the title of our paper is misleading as our studies were focused on management of NCD. The implementation barriers also focus partly on the integration with health services. We have omitted the word ‘prevention’ from the title. We realise that this is a limitation of the study. We added a sentence about this in the discussion. We mention in that part also other studies, such as the one suggested by the reviewer.

*Authors:* although the time gap was indeed larger, most studies selected were from a narrow period.

- Include the reason for excluding 1019 studies in the flowchart.

 
*Authors: *we did so. It was not possible to quantify the reasons, since this was not recorded.

- Figure 2 is difficult to interpret on its own and has not been explained in detail. It is a complex figure to follow.

*Authors:* We have simplified the figure and provided additional text to explain it better. 

- Kindly check the paper for grammatical errors. A few are listed below for your reference:
  - In the second line of study selection section; word “for” is missing *–“6 authors were responsible determining eligibility and inclusion”.*
  - In the fourth line of the same section; it should be “was” instead of “are”, as the whole paragraph is written in the past tense, except for this sentence. *“The types of studies included are randomized controlled trials, crosssectional studies, case control studies, and cohort studies.”*
  - In first line of page 4, it should be “tested” in place of “testing” – *“Although the questionnaire was not pre-testing before administration with the target group,”*

*Authors* *: Thank you for pointing out these errors. We have carefully gone through the manuscript again and re-edited for proper English.*

# Peer review: Reviewer response for version 1

**DOI:** 10.21956/wellcomeopenres.17062.r37602

**Published:** 2020-02-04

**Authors:** Harm W.J. Van Marwijk

**Affiliations:** 1 Division of Primary Care and Public Health, Brighton and Sussex Medical School, University of Brighton, Brighton, UK

This paper explores implementation issues around m-health for NCDs in low and middle-income countries from two perspectives: information in published papers and field-based knowledge by people working in this field. The link between the two is explorative and informational mostly. The conclusion aligns with a review my group did on the same topic (for an EU project).  

 Through a scoping review, publications on mHealth interventions for NCDs in LMICs were identified and assessed with the WHO mHealth Evidence Reporting and Assessment (mERA) tool. A two-stage web-based survey on implementation barriers was also performed within an NCD research network and through two online platforms on mHealth targeting researchers and implementors. This last bit might cause a bit of confusion with readers as it is basically a qualitative element of the project.

Is the work clearly and accurately presented and does it cite the current literature?

Yes

If applicable, is the statistical analysis and its interpretation appropriate?

Yes

Are all the source data underlying the results available to ensure full reproducibility?

No source data required

Is the study design appropriate and is the work technically sound?

Yes

Are the conclusions drawn adequately supported by the results?

Yes

Are sufficient details of methods and analysis provided to allow replication by others?

Yes

Reviewer Expertise:

General practice, global health.

I confirm that I have read this submission and believe that I have an appropriate level of expertise to confirm that it is of an acceptable scientific standard.
